# Granulocyte colony-stimulating factor (G-CSF) promotes spermatogenic regeneration from surviving spermatogonia after high-dose alkylating chemotherapy

**DOI:** 10.1186/s12958-016-0226-1

**Published:** 2017-01-11

**Authors:** Travis Kotzur, Roberto Benavides-Garcia, Jennifer Mecklenburg, Jamila R. Sanchez, Matthew Reilly, Brian P. Hermann

**Affiliations:** 1 Department of Biology, The University of Texas at San Antonio, One UTSA Circle, San Antonio, TX 78249 USA; 2 Departments of Biomedical Engineering and Ophthalmology, The Ohio State University, 1080 Carmack Road, Columbus, OH 43210 USA

**Keywords:** Spermatogonial stem cells, Infertility, Cancer, Late effects, Fertility preservation, Adjuvant

## Abstract

**Background:**

The lifesaving chemotherapy and radiation treatments that allow patients to survive cancer can also result in a lifetime of side-effects, including male infertility. Infertility in male cancer survivors is thought to primarily result from killing of the spermatogonial stem cells (SSCs) responsible for producing spermatozoa since SSCs turn over slowly and are thereby sensitive to antineoplastic therapies. We previously demonstrated that the cytokine granulocyte colony-stimulating factor (G-CSF) can preserve spermatogenesis after alkylating chemotherapy (busulfan).

**Methods:**

Male mice were treated with G-CSF or controls before and/or after sterilizing busulfan treatment and evaluated immediately or 10–19 weeks later for effects on spermatogenesis.

**Results:**

We demonstrated that the protective effect of G-CSF on spermatogenesis was stable for at least 19 weeks after chemotherapy, nearly twice as long as previously shown. Further, G-CSF treatment enhanced spermatogenic measures 10 weeks after treatment in the absence of a cytotoxic insult, suggesting G-CSF acts as a mitogen in steady-state spermatogenesis. In agreement with this conclusion, G-CSF treatment for 3 days before busulfan treatment exacerbated the loss of spermatogenesis observed with G-CSF alone. Reciprocally, spermatogenic recovery was modestly enhanced in mice treated with G-CSF for 4 days after busulfan. These results suggested that G-CSF promoted spermatogonial proliferation, leading to enhanced spermatogenic regeneration from surviving SSCs. Similarly, there was a significant increase in proportion of PLZF+ undifferentiated spermatogonia that were Ki67+ (proliferating) 1 day after G-CSF treatment.

**Conclusions:**

Together, these results clarify that G-CSF protects spermatogenesis after alkylating chemotherapy by stimulating proliferation of surviving spermatogonia, and indicate it may be useful as a retrospective fertility-restoring treatment.

**Electronic supplementary material:**

The online version of this article (doi:10.1186/s12958-016-0226-1) contains supplementary material, which is available to authorized users.

## Summary

The cytokine granulocyte colony-stimulating factor promotes spermatogenic regeneration from surviving spermatogonia after high-dose alkylating chemotherapy in a manner that involves enhanced proliferation of undifferentiated spermatogonia.

## Background

Currently, survival rates for childhood cancer (ages 0–14 years, all sites and races) in the US and abroad exceed 84% due to advent of more effective, life-saving cancer treatments (84.5% in US, 86% in Austria, [1, 2]). As a result of these successful oncological therapies, many survivors of childhood cancers are able to lead long, productive lives. However, these cancer survivors are often plagued by the life-long side-effects induced by the same treatments that saved their lives [3–5]. Among the most devastating of these so-called late effects (long-term side-effects) of chemotherapy and radiation treatments for cancer is male infertility [6–9]. While men and boys who have undergone puberty can ensure their future fertility by cryobanking sperm obtained from an ejaculate [10], this is not an option for pre-pubertal boys who are not yet making mature gametes.

As a result of this clinical need for fertility preservation strategies in pre-pubertal cancer patients, a number of experimental approaches have been under intense development [10–16], and specifically, to preserve fertility of pre-pubertal boys. Inherent to these strategies, however, are risks associated with invasive surgical testicular tissue retrieval, including anesthesia, infection and delays to primary disease therapy, which remain major concerns that drive the risk-benefit ratio in favor of no intervention and likelihood of permanent infertility. As an alternative, we previously identified a completely non-invasive approach to preserving male fertility after cancer treatment, using injections of the cytokine granulocyte colony-stimulating factor (G-CSF), which would obviate the need for the invasive techniques currently under development. Specifically, we recently found that G-CSF treatments in mice led to significantly better recovery of spermatogenesis after busulfan treatment than in untreated controls [17]. Serendipitously, it also appears treatment with G-CSF treatment as part of a bone marrow mobilization strategy in rhesus macaques was associated with enhanced spermatogenic recovery following busulfan chemotherapy [12, 18]. Therefore, G-CSF treatment to protect spermatogenesis from cancer treatments has the potential to revolutionize male fertility preservation in a manner that can be rapidly translated to the clinic because various forms of G-CSF are already FDA-approved (e.g., filgrastim: Neupogen® - Amgen, Granix® - Teva, Zarxio® - Novartis).

However, before G-CSF treatment can be translated to the clinic as a fertility preservation/restoration agent, more thorough examination of efficacy and mechanism of action must be undertaken. Indeed, a number of questions arose as a result of our initial study, including: 1) whether G-CSF-induced spermatogenic protection against busulfan sterilization was stable longer than the 10 weeks previously examined, 2) whether G-CSF treatment influences steady-state spermatogenesis, 3) the precise temporal window during with which G-CSF promotes spermatogenic recovery after busulfan treatment, and 4) whether G-CSF promotes proliferation of undifferentiated spermatogonia, in vivo. This present study addresses these open questions and provides additional evidence supporting the concept that treatment with G-CSF protects spermatogenesis from alkylating chemotherapies by driving proliferation of surviving undifferentiated spermatogonia. As a result, it now appears that G-CSF treatment would be most useful as a fertility-restoring adjuvant therapy to promote enhanced spermatogenic recovery and future fertility after sterilizing cancer treatments.

## Methods

### Animals

Male C57BL/6 mice were purchased from The Jackson Laboratory and maintained with ad libitum normal laboratory diet. All experiments utilizing animals were approved by the Institutional Animal Care and Use Committee of the University of Texas at San Antonio (Assurance A3592-01) and were performed in accordance with the National Research Council Guide for the Care and Use of Laboratory Animals.

### Experimental design, G-CSF and busulfan treatments

**Fig. 1:**
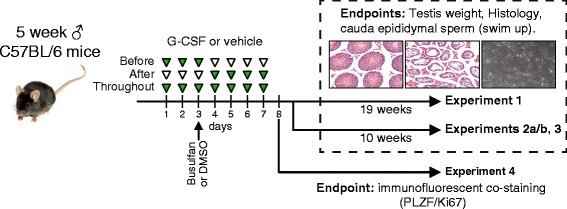
Experimental design. Four separate mouse experiments were performed to examine the effect of G-CSF on steady state spermatogenesis and spermatogenic recovery after busulfan treatment. In all experiments, 5-week old C57BL/6 males were treated with G-CSF or vehicle over the course of a 7 day period (*green* or open triangles, respectively) and given one injection of DMSO or Busulfan on day 3. The four experiments differed in the G-CSF dose, G-CSF administration duration and schedule relative to busulfan treatment, as well as the time to analysis1. Animals in Experiments 1–3 were euthanized after 10–19 weeks and effects on spermatogenesis were assessed by comparing testis weights, testis histology and cauda epididymal sperm counts (except for experiment 1). Note: mouse sperm image from MethBank: a Database of DNA Methylome Programming (http://www.dnamethylome.org/). Animals in Experiment 4 (from [17]) were euthanized 24 h following the last treatment (on day 8) and used for immunofluorescent analysis of Ki67 labeling index of PLZF+ spermatogonia

Five week old male mice were given subcutaneous injections of recombinant human granulocyte colony-stimulating factor (PeproTech) suspended in Dulbecco’s phosphate-buffered saline (DPBS; Life Technologies) containing 0.5% bovine serum albumin fraction V (BSA, MP Biomedicals) or 0.5% BSA in DPBS alone (vehicle), as described previously [17]. G-CSF dosages were either 50ug/kg/day or 125ug/kg twice daily (see Fig. 1). On the third day, mice were also given either busulfan [44 mg/kg, in dimethyl sulfoxide (DMSO); Sigma-Aldrich] or DMSO alone by a single IP injection, also as described previously [17]. In experiment 2 (a and b), three schedules of G-CSF administration were used relative to busulfan or DMSO treatment on day 3 (as described above): days 1–3 (before), days 4–7 (after), or days 1–7 (throughout). Animals were euthanized at 19 weeks (experiment 1) or 10 weeks (experiments 2 and 3 – see Fig. 1) and evaluated for spermatogenic metrics (testes weights, epididymal sperm counts, testis histololgy). In experiment 3, animals were euthanized 24 h after the last G-CSF/vehicle injection (immunofluorescent co-labeling of undifferentiated spermatogonia and Ki67; Fig. 1) as described [17].

### Testis weights and blinded histological analyses

Testes from each animal were weighed and fixed with fresh 4% paraformaldehyde, paraffin-embedded and sectioned (5 μm) and cross-sections were H&E stained. Composite tiled mosaic images of eight testis sections (≥35 μm offset between each section) were obtained at 20X magnification using an AxioImager M1 (Zeiss) and an AxioCam ICc1 (Zeiss). Round seminiferous tubule cross-sections in each image were categorized according to the degree of spermatogenesis as described previously [17] based on the most advanced germ cells present in each tubule cross section. Specifically, we counted and categorized tubules based on whether they contained complete spermatogenesis (containing all germ cell types up to and including elongating spermatids or spermatozoa), round spermatids (all germ cell types up to and including post-meiotic round spermatids, but not more advanced elongating spermatids or spermatozoa), primary spermatocytes (all germ cell types up to and including primary spermatocytes, but not more advanced germ cell types), or were empty (marked absence of germ cells, Sertoli cell-only and/or some spermatogonia). Data are reported as percentages of seminiferous tubules containing the noted categories of the most advanced germ cell types. All histological sections/images were blinded for imaging and analysis. Statistically significant differences between groups were determined by Student’s t-tests.

Seminiferous tubule diameters were calculated automatically using a digital image processing algorithm developed in MATLAB 2015b (The MathWorks, Inc) revised from a previous iteration [17] to improve characterization of challenging histological sections. Only data from round seminiferous tubule cross-sections [shape factor (4πarea/circumference^2^) values of ≥0.8] were used for subsequent analyses, an approach used previously to define roundness of isolated cells [19–21]. Tubule equivalent diameter (√(4area/π)) was calculated as the diameter of a circle with the equivalent area of each tubule cross-section.

### Sperm counts

One epididymis from each animal was used to quantify sperm counts using a swim-up technique. Briefly, each complete epididymis was minced in room temperature DBPS, incubated at 37 °C for 30 min to allow motile sperm to swim out of the ducts and sperm number per ml was determined by hemocytometer after PFA fixation.

### Immunofluorescent tissue staining

In experiment 3, testes sections were stained with antibodies against γH2A.X to identify spermatocytes (marker of DNA double-strand breaks) and with lectin peanut agglutinin (PNA) to label terminal β-galactose found on spermatid acrosomes. In experiment 4, testis sections from treated mice generated previously [17] were stained with antibodies against promyelocitic leukemia zinc-finger protein (PLZF, marker of undifferentiated spermatogonia) and Ki67 (marker of cellular proliferation), essentially as described [17, 22]. Briefly, 4% paraformaldehyde (PFA)-fixed paraffin-embedded sections were subjected to antigen retrieval in sodium citrate buffer, rinsed, and blocked in antibody diluent. Blocked sections were either labeled with antibodies against γH2A.X (2.5 μg/ml; rabbit anti-γH2A.X, ab11174, lot GR224632-3, Abcam), or concurrently with antibodies against PLZF (1 μg/ml, goat anti-PLZF IgG, AF2944, lot VUG0109121, R&D Systems) and Ki67 (2.5 μg/ml, mouse anti-human Ki67 IgG1k, Clone B56, lot 03136, BD Biosciences; [23, 24]). Antibodies were detected by indirect immunofluorescence (10 μg/ml of goat anti-rabbit IgG AlexaFluor 488, donkey anti-mouse IgG AlexaFluor 488 and/or donkey anti-Goat IgG AlexaFluor 568, all from Life Technologies), and counterstained with 1 μg/ml Hoechst 33342 (Sigma-Aldrich) to identify nuclei and/or 1 μg/ml lectin Peanut agglutinin (PNA) AlexaFluor 568 (ThermoFisher Scientific) to identify acrosomes of round and elongating spermatids. Positive immunoreactivity was validated by omission of primary antibody. Fluorescently stained sections were mounted with FluoromountG (Southern BioTech). Composite tiled mosaic images for each complete section at 20X magnification were generated using an AxioImager M1 (Zeiss) and an AxioCam MRm (Zeiss). The frequency of PLZF+ spermatogonia in round seminiferous tubule cross-sections exhibiting positive staining for Ki67 in each image was determined using NIH Image J using the Cell Counter plugin. Ki67/PLZF staining was quantified in similar numbers of round seminiferous tubule cross-sections from 4 animals per group (average number of tubules = Control 519 ± 127; Busulfan 479 ± 11; Busulfan + G-CSF 448 ± 18; not significantly different between groups).

## Results

**Fig. 2:**
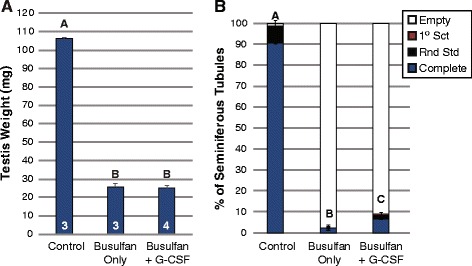
G-CSF-enhanced spermatogenic recovery after busulfan treatment is stable. Results are from animals in Experiment 1 **(a)** Testis weights (mean ± standard error). White numbers at base of bars indicate animal numbers in each group. **b** Stacked bars show the percentage of all seminiferous tubule cross-sections counted from all animals in each group which exhibit differing degrees of spermatogenesis: complete spermatogenesis (complete), up to round spermatids (Rnd Std), up to 1° spermatocytes (1° Sct), or containing no spermatogenesis (Empty or Sertoli cell-only). A, B, and C categorical notations above bars denote statistically significant differences between groups (*p* <0.05) as determined by *t-*tests. Histology results were similar to previous examples shown in our prior work [17]. The number of seminiferous tubule cross-sections evaluated per animal is shown in Additional file 1: Table S1

In our first experiment (Experiment 1, Fig. 1), male mice were separated into three groups, vehicle-treated “Control”, “Busulfan Only” (44 mg/kg), and “Busulfan + G-CSF” animals which received G-CSF (50ug/kg/day) in addition to busulfan and all were allowed to recover until 19 weeks transpired (Fig. 1). As shown previously at 10 weeks [17], busulfan treatment caused a significant decline in testis weights at 19 weeks compared with control animals, but testis weights did not differ significantly between the Busulfan Only and Busulfan + G-CSF groups (Fig. 2a). Histological examination of the testes confirmed that many seminiferous tubule cross-sections were devoid of germ cells in animals treated with busulfan (Busulfan Only and Busulfan + G-CSF groups; Fig. 2b and Additional file 1: Table S1), as compared with Control animals, in which nearly all tubule cross-sections in contained complete spermatogenesis (Fig. 2b and Additional file 1: Table S1). However, like we previously observed at 10 weeks [17], treatment with G-CSF led to significantly better spermatogenic recovery at 19 weeks than in Busulfan only group (*p* ≤ 0.0285; Fig. 2b and Additional file 1: Table S1). Specifically, there were 2.8-fold more tubule cross-sections containing complete spermatogenesis in animals treated with G-CSF compared with busulfan only (Fig. 2). These results confirm that G-CSF-induced spermatogenic protection is stable for at least twice the duration initially examined and likely originates from an effect at the level of SSCs.

**Fig. 3:**
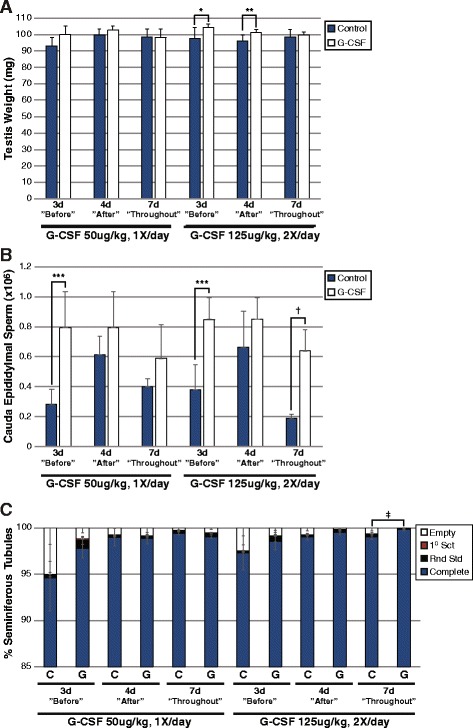
G-CSF enhances steady-state spermatogenesis in the absence of a cytotoxic insult. Results are from animals in Experiments 2a and b **(a)** Testis weights (mean ± standard error). Significant differences were determined by a Student’s *t*-test and are noted by asterisks above noted bars (*p ≤ 0.057, **p ≤ 0.083). **b** Cauda epididymal sperm counts (swim-up, mean ± standard error). Notation above bars denotes a statistically significant difference between groups (***p ≤0.03, #*p* ≤0.03, †*p* ≤0.006) as determined by *t-*test. **c** Stacked bars show the percentage of all seminiferous tubule cross-sections counted from all animals in the Control (C) or G-CSF alone (G) groups which exhibit differing degrees of spermatogenesis: complete spermatogenesis (complete), up to round spermatid spermatids, up to 1° spermatocytes, or containing no spermatogenesis (empty or Sertoli cell-only). Notation above bars denotes a statistically significant difference between groups (‡*p* ≤0.002) as determined by *t-*test. The number of seminiferous tubule cross-sections evaluated per animal is shown in Additional file 1: Table S1

To determine if G-CSF treatment had an effect on steady state spermatogenesis, in Experiment 2 (Fig. 1), we compared control animals that received vehicle injections or “G-CSF Only” animals which received one of two G-CSF doses (50ug/kg/day – Exp 2a or 125ug/kg twice daily – Exp 2b) for 3 days, 4 days or 7 days (Fig. 1). The higher G-CSF dose (125ug/kg, twice daily; 250ug/kg/day) was chosen based on the effective dose required for hematopoietic stem cell mobilization in mice [25–27]. Animals received escalating schedules of 3, 4 or 7 days of G-CSF (or control) injections in order to match the treatment course in Experiment 3 (in which animals received either Busulfan or Busulfan + G-CSF treatment). Testis weights were unchanged in animals treated with 50ug/kg/day G-CSF compared with controls (Fig. 3a). Modest increases in testis weight that were suggestive of statistical significance were observed with either 3 days or 4 days of high-dose G-CSF treatment (*p ≤0.057 and **p ≤0.083, respectively; Fig. 3a). Compared with controls, cauda epididymal sperm counts were also significantly higher in animals receiving 3 days of G-CSF treatment (50ug/kg/day or 250ug/kg/day) or 7 days of high-dose G-CSF treatment (***p ≤0.03 and †*p* ≤0.006, respectively; Fig. 3b). As expected, G-CSF treatment with 50ug/kg/day did not significantly alter the proportion of seminiferous tubules that contained complete spermatogenesis (Fig. 3c). Only testes of animals treated for 7 days with the high G-CSF dose exhibited a significantly increased proportion of seminiferous tubules containing complete spermatogenesis (‡*p* ≤0.002; Fig. 3c), although the absolute difference was marginal (99.05% vs. 99.85% complete spermatogenesis, respectively). Diameters of round seminiferous tubules from mice that received 50ug/kg/day G-CSF for 3 days were significantly smaller than controls (148 ± 3 μm vs.168 ± 3 μm, respectively; Additional file 2: Figure S1A), while all other groups were not significantly different (Additional file 2: Figure S1A), indicating equivalent extent of spermatogenesis among seminiferous tubules. Together, these data suggest that G-CSF may modestly enhance steady-state spermatogenesis in the absence of a cytotoxic insult.

**Fig. 4:**
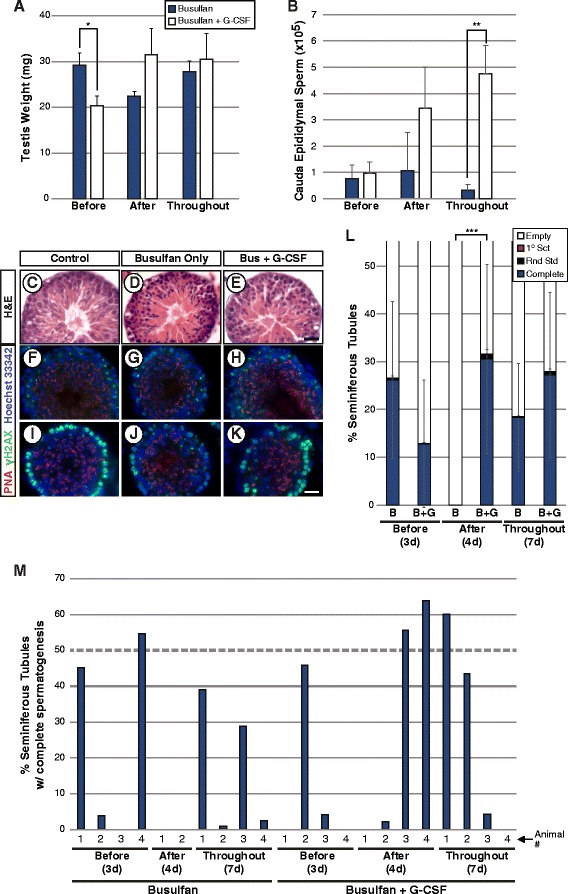
G-CSF exacerbates spermatogenic loss before busulfan treatment but enhances recovery after busulfan treatment. Results are from animals in Experiment 3. **a** Testis weights (mean ± standard error). Significant differences were determined by a Student’s *t*-test and are noted by asterisks above noted bars (*p ≤0.006). **b** Cauda epididymal sperm counts (swim-up, mean ± standard error; **p ≤0.002). Micrographs of individual seminiferous tubule cross-sections demonstrating complete spermatogenesis **(c-e)** stained with H&E, or with antibodies against **(f-h)** γH2A.X (spermatocyte marker) and **(i-k)** lectin peanut agglutinin (PNA; spermatid marker) are shown from representative sections of testes from **(c, f, i)** Control, **(d, g, j)** Busulfan only group and **(e, h, k)** Busulfan + G-CSF group. Scale bars = 20 μm. **l** Stacked bars show the percentage of all seminiferous tubule cross-sections counted from all animals in each group which exhibit differing degrees of spermatogenesis: complete spermatogenesis (complete), up to round spermatid spermatids, up to 1° spermatocytes, or containing no spermatogenesis (empty or Sertoli cell-only), ***p ≤0.086). The number of seminiferous tubule cross-sections evaluated per animal is shown in Additional file 1: Table S1. **m** The proportion of seminiferous tubule cross-sections containing complete spermatogenesis in each individual animal (number indicated below x-axis), grouped by treatment group

We performed a third experiment in order to determine when G-CSF promotes spermatogenic recovery relative to busulfan treatment, before and/or after the cytotoxic insult (Fig. 1). In Experiment 3, busulfan only animals were compared to busulfan + G-CSF animals which received high-dose G-CSF (125ug/kg, twice daily) on three different schedules: for 3 days ending on the day of busulfan treatment (Before), for four days starting on the day after busulfan treatment (After), and for all seven days (Throughout; Fig. 1) and again evaluated at 10 weeks. Testis weights of animals receiving G-CSF before busulfan were significantly reduced compared with busulfan only controls (*p ≤0.006; Fig. 4a), and while those which received G-CSF after busulfan or throughout the week were higher than controls, the differences were not statistically significant. Cauda epididymal sperm counts from animals receiving G-CSF either before or after busulfan were not significantly different from busulfan only controls, but sperm counts for animals treated with G-CSF for the entire week were significantly higher (**p ≤0.002; Fig. 4b), in agreement with previous results [17].

We observed seminiferous tubules in animals treated with Busulfan alone and animals that also received G-CSF treatment that contained apparently normal spermatogenesis that was similar to control animals that did not receive any busulfan or G-CSF (Fig. 4c-k). These seminiferous tubules with complete spermatogenesis appeared histologically normal (Fig. 4c-e), but were significantly smaller in diameter than those from control testes (Additional file 2: Figure S1B). There were no differences in diameters of spermatogenesis-containing seminiferous tubules between busulfan-treated animal groups with or without G-CSF treatment (Additional file 2: Figure S1B). As expected, tubules with complete spermatogenesis also exhibited punctate staining for gamma-H2A.X in pachytene spermatocytes (Fig. 4f-h) and intense diffuse nuclear gamma-H2A.X staining in preleptotene-through early zygotene spermatocytes (Fig. 4i-k; [28]), and intense lectin peanut agglutinin staining on acrosomes of round and elongating spermatids (Fig. 4f-k; [29, 30]). Testes of animals treated with G-CSF before busulfan tended to have fewer seminiferous tubules with complete spermatogenesis (Fig. 4l), in line with testis weights. However, G-CSF administration after busulfan treatment led to an increase in the proportion of seminiferous tubules with complete spermatogenesis at a level that was suggestive of significance (***p ≤0.086; Fig. 4e, f, i). G-CSF administration throughout the week exhibited a trend towards increased complete spermatogenesis (Fig. 4c, d, i) in agreement with previous results [17]. The lack of statistical significance at the level of *p* <0.05 was at least partly due to the pronounced variability in efficiency of busulfan-induced spermatogenic loss (compare three busulfan alone groups, Fig. 4l). Examination of the results from each individual animal, however, demonstrated that only one of ten animals that received Busulfan alone recovered complete spermatogenesis in at least 50% of seminiferous tubule cross-sections (10%), while 2/4 animals in the “after” Busulfan + G-CSF group recovered spermatogenesis to this extent and both animals in this group exhibited >55% seminiferous tubules containing complete spermatogenesis (Fig. 4m). Reciprocally, none of the animals in “before” Busulfan + G-CSF group recovered spermatogenesis exceeding to 50% or greater (0%; Fig. 4m). Thus, while it is important to note that we observed significant variability in the effect of busulfan, these results demonstrate that G-CSF exacerbated spermatogenic loss when given before busulfan treatment and enhanced spermatogenic recovery from surviving spermatogonia after busulfan treatment.

**Fig. 5:**
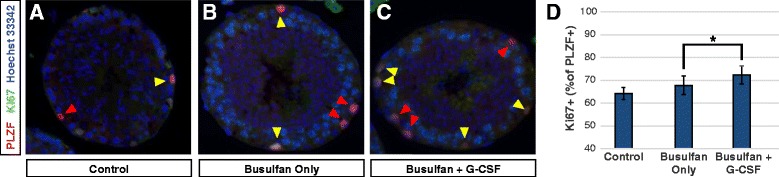
G-CSF treatment promotes proliferation of PLZF+ spermatogonia after busulfan treatment. Result are from mice in Experiment 4. **a-d** Testis sections were co-stained for PLZF (*green*) and Ki67 (*red*) to determine the percentage of PLZF+ spermatogonia that were proliferating (Ki67+). Red arrowheads point to PLZF+/Ki67- cells and yellow arrowheads mark PLZF+/Ki67+ cells. The asterisk notation above bars denotes a statistically significant increase (*p* ≤0.0352) in the Ki67 labeling index of PLZF+ spermatogonia as determined by Student’s *t*-test. Scale bars = 50 μm

To address the possibility that G-CSF promotes spermatogenic regeneration by stimulating proliferation of surviving undifferentiating spermatogonia after busulfan treatment, we examined Ki67 labeling of PLZF+ undifferentiated spermatogonia in testes of mice analyzed 24 h after the last G-CSF or vehicle treatment (tissues were from animals reported previously [17]). The Ki67 protein is an established marker of cellular proliferation because its expression is observed only during G1, S, G2 and M phases of the cell cycle and not during G0 phase [31]. PLZF [32, 33] is a transcription factor expressed by undifferentiated spermatogonia in rodents (A_single_, A_paired_ and A_aligned4–16_ spermatogonia [34]). As expected, busulfan-treated mice that also received G-CSF had significantly higher proportion of PLZF+ undifferentiated spermatogonia that were co-labeled for Ki67 (proliferating) than busulfan only animals (*p* ≤0.035; Figure 5a-d). Thus, the increased numbers of PLZF+ spermatogonia previously reported after busulfan treatment induced by G-CSF [17] is accompanied by an increase in proliferative index.

## Discussion

Preserving and restoring male fertility after chemotherapy and radiation treatments for cancer and other non-malignant disorders has received significant scientific attention for more than a decade, and in particular, fertility preservation among pre-pubertal boys who are not yet making sperm has been a major research focus around the world [6, 7, 10, 11, 16, 35–37]. We previously described an alternative adjuvant strategy using injections of the cytokine G-CSF at around the time of chemotherapy/radiation treatment to enhance spermatogenic recovery and potentially preserve fertility [17]. Here, we provide additional evidence of the stability of the protective effect of G-CSF on spermatogenesis after chemotherapy and suggest that the mode of G-CSF action is by promotion of spermatogonial proliferation, leading to enhanced spermatogenic regeneration from surviving SSCs.

Notably, though, the efficacy of busulfan sterilization (revealed by the proportion of tubule cross-sections with spermatogenesis) was variable both between studies and within the current study. For instance, we previously reported that ~9.5% of tubule cross-sections contained any degree of spermatogenesis [17], yet we observed only 2.5% of tubules contained spermatogenesis in experiment 1 (see Fig. 2). Likewise, in experiment 3, the proportion of seminiferous tubules containing spermatogenesis in busulfan only animals ranged from 0% (after group) to 26.6% (before group), with an average of 18.7% ± 7.7%, demonstrating considerable intra-experiment variation. While it is not possible to directly compare the results from different experimental groups because each received different treatment regimens, future studies might explore methods to improve reliability of busulfan sterilization, alternative agents (e.g., cyclophosphamide), or use of irradiation. Highly variable busulfan pharmacokinetics in human patients, even intra-individually, is well known [38–41]. Therefore, although we took great care to ensure accurate busulfan dosing and employed an inbred mouse strain to reduce the influence of genetic modifiers on the experimental outcomes, differences in busulfan pharmacokinetics between individual animals could have led to the variability in busulfan efficacy we observed. These results also highlight that the danger of assuming consistent efficacy of busulfan sterilization. Despite variability of the primary sterilizing agent (busulfan), G-CSF treatment appeared to enhance spermatogenic recovery thereafter.

Since the receptor for G-CSF, colony-stimulating factor 3 receptor (CSF3R), has been previously detected on the cell surface of cultured THY-1 Cell Surface Antigen + (THY1+) undifferentiated spermatogonia [17], it was possible that G-CSF may act directly on undifferentiated spermatogonia and have a role in promoting normal steady state spermatogenesis. Results from experiment 2 demonstrated modest enhancement in all spermatogenic parameters examined in animals exposed only to G-CSF (testis size, caudal sperm counts, proportion of seminiferous tubules containing complete spermatogenesis), suggesting that G-CSF may drive maximal spermatogenic output. It is not surprising that the absolute extent of spermatogenesis was only modestly influenced by administering exogenous G-CSF because the spermatogenic ceiling is established by Sertoli cell number, which dictates the number of available niches for spermatogonial stem cells (SSCs) and extent of trophic support for differentiating spermatogenic cells [42–49]. Postnatal mouse Sertoli cells proliferate only until about 15–20 days postpartum [50, 51], and since Sertoli cell number establishes the spermatogenic ceiling, the extent of spermatogenesis could not be enhanced substantially by G-CSF in the five week old mice used in this study.

G-CSF binding to its receptor promotes cellular proliferation in a variety of tissues, both in vivo and in vitro [52–56]. Therefore, if G-CSF promotes proliferation of undifferentiated spermatogonia expressing the CSF3R protein, the susceptibility of the germline to a cytotoxic insult, such as the alkylating agent busulfan, should differ based on proliferative drive by G-CSF. In the present study, we observed elevated spermatogenic susceptibility to busulfan (e.g., smaller testes and fewer tubules with spermatogenesis) when G-CSF was given before busulfan, consistent with increased spermatogonial proliferation before cytotoxic insult. Likewise, mice had larger testes with more spermatogenesis when G-CSF was given only after busulfan, also consistent with increased spermatogonial proliferation leading to enhanced regeneration from surviving spermatogonia. Animals receiving G-CSF both before and after busulfan exhibited an intermediate, but positive effect on spermatogenesis consistent with previous studies [17]. Results of a previous study in which a moderate dose of G-CSF (100ug/kg/day) was administered for 3 days prior to sub-sterilizing (5Gy) gamma irradiation and in which a seemingly protective effect of G-CSF on repopulation of seminiferous tubules was reported [57] appeared to conflict with results of the present study. But, this apparent discrepancy could arise from any of a number of differences in the experimental design and treatment schedule, including use of a sub-sterilizing radiation dose (vs. high-dose busulfan), different metrics used in establishing repopulation (≥3 differentiating Type-B spermatogonia in a tubule cross-section) or the short (3 week) time to analysis (vs ≥10 weeks). Future studies to more extensively evaluate the time-course of G-CSF-induced spermatogenic regeneration and test its efficacy in the context of other cytotoxic agents would help to resolve these discrepancies.

While it would not have been informative to examine spermatogonial proliferation directly in the animals from experiment 3, since the effects of G-CSF would have been realized only at the time of treatment, we did revisit the proliferative index among undifferentiated spermatogonia from mice treated with G-CSF and analyzed acutely thereafter [17]. A small, but statistically significant increase in Ki67+ labeling index of PLZF+ spermatogonia in animals treated with G-CSF in addition to busulfan is consistent with the potential role of G-CSF in promoting proliferation of undifferentiated spermatogonia and results of previous experiments demonstrating an increase in PLZF+ spermatogonial numbers with no change in apoptosis [17]. Ki67 is a protein antigen that is specifically expressed during G1, S, G2, M cell cycle phases of proliferating cells and essentially absent in cells that are arrested in the G0 phase [31], and thus, is recognized as an excellent marker of cell proliferation. However, even well-controlled antibody staining experiments can lead to spurious results. Thus, future studies in support of the Ki67+ labeling index reported here could examine S-phase labeling index by incorporation of thymidine analogs (e.g., BrdU, EdU, etc.), both in vivo and in vitro.

Like previous studies in both males and females [17, 57, 58], G-CSF improved the extent of gametogenesis after busulfan chemotherapy in our present studies. However, despite temporal optimization and increased G-CSF doses, spermatogenic recovery after busulfan treatment was still incomplete in our studies. This consistent result begs the question whether the extent of spermatogenic regeneration induced by G-CSF after a cytotoxic insult will be sufficient to promote recovery of fertility. Such an outcome would make G-CSF treatment an effective clinical adjuvant therapy for promoting male fertility restoration after sterilizing therapies. Future studies will be needed to determine if G-CSF effectively restores male fertility after sterilizing chemotherapy insults. With a burgeoning population of cancer survivors, some of whom will have received G-CSF for chronic neutropenia after treatment for their primary disease, it may be possible to interrogate whether G-CSF promotes fertility restoration in human patients. Therefore, it is possible that patients who receive adjuvant G-CSF to address a common hematopoietic side-effect of cancer therapy, both as children or in adulthood, may be less likely to remain infertile after a sterilizing therapy than patients who receive only chemotherapy/radiation for their primary disease. It is important to note, though, that the studies examining G-CSF as a protective agent performed to date have utilized adult animals, and thus, the applicability to prepubertal cancer patients will first need to be established by examining the influence of G-CSF on spermatogenic recovery in immature animals. Thus, it would be premature to apply G-CSF clinically as a means of protecting or restoring fertility in humans until such studies have been completed. Ultimately, though, G-CSF may serve as another instrument to address male infertility as one of the most common side-effects of lifesaving treatments for cancer.

## Conclusions

Granulocyte colony-stimulating factor (G-CSF) appears to promote proliferation of undifferentiated spermatogonia, which leads to a modest enhancement of spermatogenic regeneration from surviving spermatogonia after high-dose alkylating chemotherapy. G-CSF treatment alone also enhances spermatogenic parameters, suggesting a role in steady-state spermatogenesis.

## Additional files

Additional file 1: Table S1.﻿﻿﻿ This spreadsheet presents the raw results of histological analyses from all experiments. Columns list the numbers and percentages of seminiferous tubules that fall into each category as well as the number of round seminiferous tubules counted and the number of any tubules excluded from analyses. Rows present data for each animal grouped by experimental group and experiment. The summary portion of this table (at bottom) lists averages by experimental group and experiment from the raw data presented above. (XLSX 24 kb)
Additional file 2: Figure S1. Seminiferous tubule diameters of animals in Experiments 2 and 3. Results are from mice in **(A)** Experiment 2 and **(B)** Experiment 3. Round seminiferous tubules were defined as having a shape factor of ≥0.8 (shape factor = 4πarea/circumference2), where a value closer to 1 is a more perfect circle. Morphometrics were reported for only seminiferous tubule cross-sections containing complete spermatogenesis. Shown are tubule equivalent diameters (equivalent diameter = √(4area/π) which provides the diameter of a circle with the equivalent area as the noted tubule cross-section. All values are average ± SEM. Labels above bars signify statistically-significant differences between groups as determined by student’s *t*-test (* *p* <0.001 control vs. G-CSF; ** *p* <0.01 control vs. Busulfan and/or Busulfan + G-CSF). (PDF 406 kb)

## Abbreviations

1° Sct: Primary spermatocytes, male germ cells in Meiosis I. Bromodeoxyuridine, uridine analog used for S-phase labeling.
BSA: Bovine serum albumin
CMYK: Cyan-magenta-yellow-black, four color space for printing.
CSF3R: Colony-stimulating factor 3 receptor, receptor for G-CSF
DMSO: Dimethyl sulfoxide, solvent for busulfan.
DPBS: Dulbecco’s phosphate-buffered saline
EdU: 5-ethynyl-2’-deoxyuridine, uridine analog used for S-phase labeling.
G-CSF: Granulocyte colony-stimulating factor (aka: CSF3)
Gy: Gray, SI unit of absorbed radiation.
H&E: Hematoxylin and eosin, histological stain.
KI67: Ki-67 protein is a cellular marker for proliferation
PFA: Paraformalehyde, fixative.
PLZF: Promyelocitic leukemia zinc-finger protein (aka: ZBTB16), a cellular marker of undifferentiated spermatogonia.
PNA: Peanut agglutinin, marks spermatids and spermatozoa.
RGB: Red-green-blue, three color space for printing.
Rnd Std: Round spermatids, post-meiotic male germ cells.
SSC: Spermatogonial stem cell
THY1+: THY-1 Cell Surface Antigen (AKA: CD90), used for enriching undifferentiated spermatogonia.
γH2A: X H2A histone family, member X, phosphorylated on serine 139, marker of DNA double-strand breaks.
