# Supplementary figures and images for: Granulocyte colony-stimulating factor (G-CSF) promotes spermatogenic regeneration from surviving spermatogonia after high-dose alkylating chemotherapy
Source: Reprod Biol Endocrinol. 2017 Jan 11;15:7. doi: 10.1186/s12958-016-0226-1 (PMC5225630; doi:10.1186/s12958-016-0226-1)

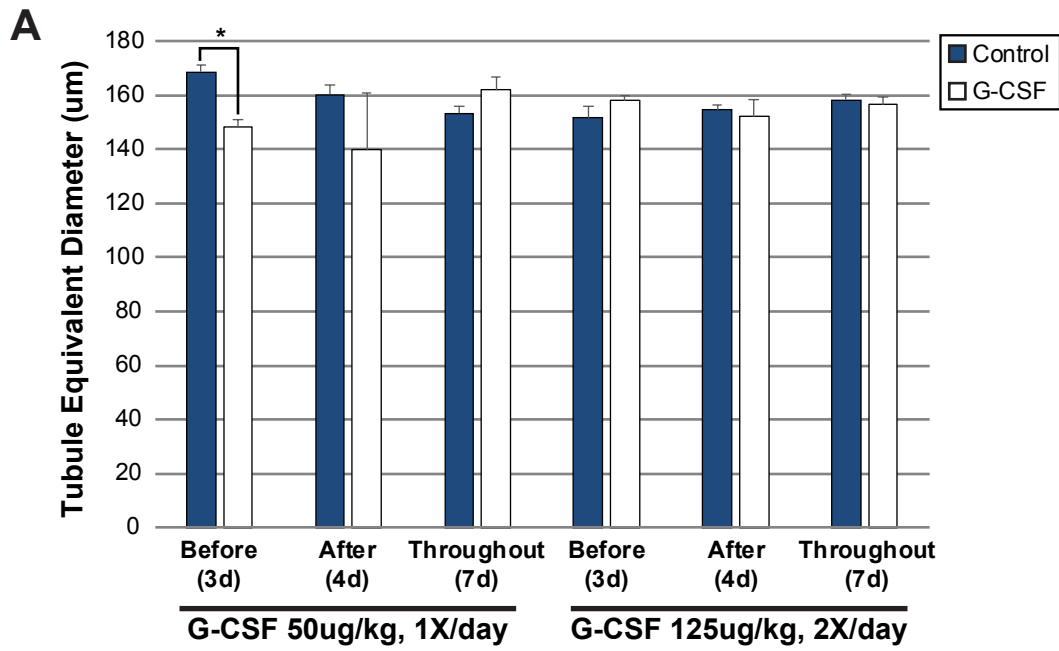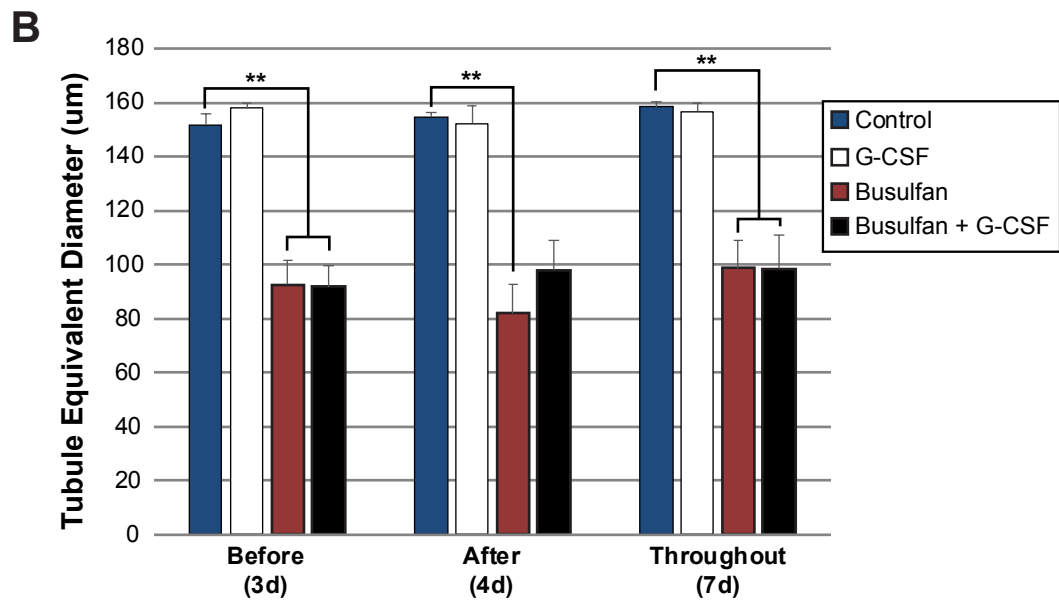

Supplement: Additional file 2: Figure S1. — Seminiferous tubule diameters of animals in Experiments 2 and 3. Results are from mice in (A) Experiment 2 and (B) Experiment 3. Round seminiferous tubules were defined as having a shape factor of ≥0.8 (shape factor = 4πarea/circumference2), where a value closer to 1 is a more perfect circle. Morphometrics were reported for only seminiferous tubule cross-sections containing complete spermatogenesis. Shown are tubule equivalent diameters (equivalent diameter = √(4area/π) which provides the diameter of a circle with the equivalent area as the noted tubule cross-section. All values are average ± SEM. Labels above bars signify statistically-significant differences between groups as determined by student’s t-test (* p <0.001 control vs. G-CSF; ** p <0.01 control vs. Busulfan and/or Busulfan + G-CSF). (PDF 406 kb) [file 12958_2016_226_MOESM2_ESM.pdf]
